# Pyrazole derivatives ameliorate synovial inflammation in collagen-induced arthritis mice model via targeting p38 MAPK and COX-2

**DOI:** 10.1007/s00210-024-03290-6

**Published:** 2024-07-29

**Authors:** Ahlam M. Abdallah, Amany H. Abdel Naiem, Salama R. Abdelraheim, Omar M. Mohafez, Hend M. Abdelghany, Sahar A. Elsayed, Wafaey Gomaa, Heba Marey

**Affiliations:** 1 Department of Medical Biochemistry, Faculty of Medicine, Minia University, Minia, 61511 Egypt; 2 Department of Biochemistry, Faculty of Pharmacy, Al-Azhar University, Assiut Branch, Assiut, 71524 Egypt; 3 Department of Rheumatology and Rehabilitation, Faculty of Medicine, Sohag University, Sohag, 82524 Egypt; 4 Department of Pathology, Faculty of Medicine, Minia University, Minia, 61511 Egypt

**Keywords:** CIA, p38 MAPK, Pyrazole derivatives, COX-2

## Abstract

The type II collagen-induced arthritis (CIA) model and human rheumatoid arthritis exhibit similar characteristics. Both diseases involve the production of inflammatory cytokines and other mediators, triggering an inflammatory cascade linked to bone and cartilage damage. Recently, new pyrazole compounds with various pharmacological activities, including antimicrobial, anticancer, anti-inflammatory, and analgesic agents, have been reported. Our aim is to evaluate the therapeutic effectiveness of two newly synthesized pyrazole derivatives, M1E and M1G, in reducing inflammation and oxidative stress in a mouse model of collagen-induced arthritis. Arthritis was induced in DBA/1J mice, and the therapeutic effect of the M1E and M1G is assessed by measuring the arthritic index, quantifying the expression of inflammatory genes such as p38 MAPK, COX-2, IL1β, MMP3, and TNF-α using real-time PCR and analyzing protein expression using western blotting for phosphorylated p38 MAPK and COX-2. Oxidative stress markers and hind paws joint histopathology were also evaluated. Treatment with the two pyrazole derivatives significantly (*p* < 0.001) improved the arthritic score; downregulated the expression of inflammatory genes p38 MAPK, COX-2, IL1β, MMP3, and TNF-α; and reduced the protein expression of phosphorylated p3  MAPK and COX-2. In addition, both compounds ameliorated oxidative stress by increasing the activities of SOD and reducing the formation of MDA in the paw tissue homogenates. Both M1E and M1G significantly (*p* < 0.001) improved the pathological features of synovitis. The pyrazole derivatives, M1E and M1G, significantly reduced the arthritic score and the inflammatory cytokine expression, improved synovitis histopathology, and ameliorated oxidative stress in the CIA mice model.

## Introduction

The type II collagen-induced arthritis (CIA) model and human rheumatoid arthritis exhibit similar pathologic and physiological characteristics because collagen type II (CII) might act as an autoantigen in joints (Park et al. 2021). Both diseases involve the production of a series of complex inflammatory cytokines and other soluble mediators by immune cells (lymphocytes and macrophages) and synovial tissues, triggering a pathogenic inflammatory cascade. These inflammatory responses are linked to the severity of bone damage and cartilage destruction as RA progresses (Lubberts and van den Berg 2013). Therefore, the CIA model is often used as an animal model for RA to demonstrate the pathological mechanisms that are relevant to human RA and to identify potential therapeutic agents (Zhang et al. 2013). CIA occurs in two phases: the first phase is acute and lasts from 0 to 10 days and is caused by the release of histamine, serotonin, and PG by immune cells, followed by the second phase, which is chronic and lasts from 11 to 28 days. During this stage, there is a disruption of pro-/anti-inflammatory responses, leading to synovitis, inflammatory cell infiltration, hypertrophy, cartilage degradation, joint dysfunction, and bone erosion (Foyet et al. 2015).

P38 mitogen-activated protein kinase (MAPK) is a crucial regulator for producing pro-inflammatory cytokines. When p38 MAPK is phosphorylated, it triggers the transcriptional activation of the pro-inflammatory TNF-α and IL-1 (Genovese et al. 2011). IL1β and TNF-α trigger RA progression via mediators like cyclooxygenase-2 (COX-2), which increases prostaglandin E2 (PGE2) production, leading to synovial inflammation and matrix metalloproteinases (MMPs) formation (Nee et al. 2007). These cytokines cause joint swelling, inflammation, cartilage, and bone erosion via the formation and activation of osteoclasts. Thus, reducing these pro-inflammatory cytokines could be an effective therapeutic approach for RA (Moon et al. 2010).

Pyrazoles are one of the most notable five-membered nitrogen-containing heterocycles extensively used in various biological activities such as antimicrobial, anticancer (Ansari et al. 2017), anti-inflammatory, and analgesic compounds (Datar and Jadhav 2015). In the previous study, the team of our lab (Elbastawesy et al. 2020) has synthesized pair of new pyrazole derivatives, compounds M1E or 6a ((E)-4-(2-benzylidenehydrazinyl) quinolin-2(1H)-one) and M1G or 6g (4-(2-(4-(dimethylamino) benzylidene)hydrazinyl)quinolin-2(1H)-one), with potential anti-inflammatory and analgesic characteristics, as a part of the ongoing research interest to find safer, more effective anti-inflammatory agents to treat rheumatoid arthritis. Our current study focused on evaluating the ability of the newly synthesized compounds M1E and M1G to reduce articular inflammation and oxidative stress biomarkers in mice model of collagen-induced arthritis and to gain insight into their mechanism(s) of action.

## Materials and methods

The following reagents were used: complete Freund’s adjuvant (CFA, Sigma-Aldrich, F5881), incomplete Freund’s adjuvant (IFA, Sigma-Aldrich, F5506), and indomethacin (Sigma-Aldrich Cat no: 405268), and immunization-grade chick type II collagen was prepared according to Miller (1972) and Herbage et al. (1977) protocols from chicken sternal cartilage. The pyrazole derivatives, M1E and M1G, were synthesized as previously described (Elbastawesy et al. 2020) and correspond to compounds 6a and 6g. Malondialdehyde (MDA) and superoxide dismutase (SOD) purchased from Bio-Diagnostic Co. HERA SYBR® Green RT-qPCR Kit, One-Step Kit (Willowfort Co., Cat no: WF10303002, UK). The mouse primers utilized for real-time PCR were as follows: p38 MAPK (MP208343), COX-2 (MP211827), IL1β (MP206724), MMP3 (MP207901), TNF-α (MP217748), and β-actin (MP200232) (OriGene Technologies, Inc., USA). Phosphorylated P38 MAPK polyclonal antibody (Cat no: E-AB-21027), COX-2 polyclonal antibody (Cat no: E-AB-70031), anti-β-actin antibody (Cat. no: E-AB-40517), and anti-rabbit IgG alkaline phosphatase-conjugated secondary antibody (Cat no: E-AB-1047) were purchased from Elabscience, USA, via the local distributing agent and used according to the manufacturing protocol.

### Animals and establishment of the model

Forty-five male DBA/1J mice (weighing 25–35 g) at 10–12 weeks of age were purchased from the Nile Centre for Experimental Research and maintained under pathogen-free conditions. They were housed in a room with an ambient temperature of 22 to 25 °C and a 12-h light/dark cycle. All animal care and procedures followed the National Institutes of Health Guidelines laboratory for animal care and use. Mice were housed in stainless steel cages and given unlimited access to food and water for the course of the study. A total of 45 DBA/1J mice were divided into two groups. Group 1: Five mice were designated healthy controls and received no injections. The remaining animals (40) were used to induce polyarthritis. Mice were immunized with type II collagen that was dissolved in 0.1 M acetic acid (4 mg/ml) at 4 °C and then emulsified with an equal volume of complete Freund’s adjuvant at day 0. Each mouse was immunized intradermal with 100 µl of emulsion at the base of the tail. The same method was used to inject incomplete Freund’s adjuvant and chicken type II collagen (100 µl/mouse) emulsion in a second intradermal tail booster injection on the 21st day following the initial immunization (Yanaba et al. 2007 and Lo et al. 2008). One week following the second immunization, by day 28, the onset of the disease activity was marked by redness and swelling of digits. Seven mice did not exhibit the usual symptoms of arthritis and were excluded from our study, while three mice from the CIA model group died during the experiment.

### Therapeutic approach

On the 29th day of initializing immunization, mice with CIA were divided into six groups of five mice each as follows: The arthritic group consists of mice with CIA without treatment as positive control. Indomethacin group: received 1 mg/kg indomethacin daily subcutaneously for 5 days as standard therapy as described by Park et al. (2021). The dose of M1E and M1G was chosen based on preliminary experiments using different dosages (1, 3, 5, or 10 mg/kg) over different time frames (5, 10, and 15 days). We found that a dose of 5 mg/kg for 15 days had a significant anti-inflammatory effect, reducing paw swelling, redness, and inflammation in collagen-induced arthritic mice. M1E group: received 5 mg/kg daily subcutaneously for 5 or 15 days. M1G group: received 5 mg/kg daily subcutaneously for 5 or 15 days. The solvent of M1E and M1G is 50 µl DMSO dissolved in 950 µl saline. On day 44, mice were anesthetized and sacrificed through cervical dislocation. The hind limbs were removed for biochemical investigations such as real-time PCR, western blot analysis, SOD, MDA, and pathological evaluation.

### Assessment of the severity of arthritis

Arthritic indices of the mice were assessed macroscopically every 3 days in all four limbs during the experiment. The morphological aspects of polyarthritis, such as inflammation, redness, and paw swelling, were visually recorded and scored from 0 to 4 for each limb by visual inspection and agreement by two investigators. The arthritis score was determined using the method outlined by Xu et al. (2019). A score of 0 was given if there was no erythema and swelling in the limb. A score of 1 was given if there was erythema and mild swelling in a single digit or toe joint. A score of 2 was given if there was erythema and swelling in more than one digit or erythema and mild swelling extending from the tarsals to the ankle. A score of 3 was given if there was erythema and swelling in the entire paw. Finally, a score of 4 was given if there was severe swelling and erythema in the entire paw, resulting in limb ankyloses and immobility. The disease activity score was the sum of the disease severity scores for each limb, with a maximum score of 16 for each mouse.

### Quantitative real-time reverse transcriptase-polymerase chain reaction (qRT-PCR)

Total RNA was extracted from ankle joint homogenates after homogenization in 1 ml of triazol reagent according to the manufacturer’s instructions. PCR reactions were prepared to determine mouse p38 MAPK, COX-2, IL1β, MMP3, and TNF-α by HERA SYBR® Green RT-qPCR Kit, One-Step Kit according to the manufacturer’s instructions. Samples were placed in a thermal cycler (Applied Biosystems StepOne™ Real-Time PCR System Thermal Cycling Block 7500 Fast, Thermo Fisher Scientific, Singapore, Ltd, USA). Real-time qRT-PCR was used to quantify gene expression that was normalized to the expression of the housekeeping gene, β-actin, using the 2^−ΔΔCT^ method to analyze the fold change in mRNA expression levels of target genes in samples, according to the published method (Abd-Allah et al. 2014). The sequences of the primers used are as follows: p38MAPK forward: GGTCATTCAGGCATCCGAGAAG, reverse: CAGAAGTCCACGAGTTCCTGCT, COX-2 forward: GCGACATACTCAAG CAGGAGCA, reverse: AGTGGTAACCGCTCAGGTGTTG; IL1β forward: TGGACCTTCCAG GATGAGGACA, reverse: GTTCATCTCGGAGCCTGTAGTG; MMP3 forward: CTCTGGAACCTGAGACATCACC, reverse: AGGAGTCCTGAGAGATTTGCGC; TNF-α forward: GGTGCCTATGT CTCAGCCTCTT, reverse: GCCATAGAACTGATGAGAGGGAG; β-actin forward: CATTGCTGACAGGATGCAGAAGG, reverse: TGCTGGAAGGTG GACAGTGAGG.

### Western blot analysis

As described previously, protein samples of ankle joint homogenates were resolved by 10% SDS–polyacrylamide gel electrophoresis (Mohamed et al. 2021). The antibodies used were phosphorylated p38 MAPK polyclonal antibody (dilution 1:2000), COX-2 polyclonal antibody (dilution 1:2000), and anti-β-actin antibody (dilution 1:3000). The membranes were incubated for 1 h with goat anti-rabbit IgG alkaline phosphatase-conjugated secondary antibody (dilution 1:3000). The BCIP/NBT substrate detection kit was used, and the generated bands were analyzed using ImageJ® software (National Institutes of Health, Bethesda, USA) for β-actin.

### Oxidative stress markers in paw tissue homogenates

The concentration of malondialdehyde (MDA) in 10% w/v mouse paw tissue homogenate was evaluated using spectrophotometry by measuring thiobarbituric acid reactive substance (TBARS), and the absorbance was determined at 534 nm. Superoxide dismutase (SOD) activity was measured in 10% w/v mouse paw tissue homogenate. Tissue protein concentration was determined according to Lowry’s method (Lowry et al. 1951) in which bovine serum albumin was used as standard (Akhtar et al. 2016). SOD was assessed by colorimetric method, and absorbance was determined at 560 according to the protocol of Nishikimi et al. (1972).

### Pathological assessment of synovitis

The hind paws were fixed in 10% buffered formalin and decalcified in 15% EDTA for histologic study of synovial membranes and assessment of the inflammatory changes. The hind paws were dissected into 5-µm sagittal serial tissue slices for paraffin embedding, and they were then stained with hematoxylin and eosin (H&E) using standard techniques (Endale et al. 2013). The synovial membranes were graded on H&E-stained slides based on the three synovial membrane parameters, synovial lining cell layer, stroma cell density, and inflammatory infiltrate, with scores ranging from none (0), slight (1), and moderate (2) to strong (3). The following are the interpretations of the parameter values: 0–1 means no synovitis, 2–4 means low-grade synovitis, and 5–9 means high-grade synovitis (Krenn et al. 2006).

### Statistical analysis

To analyze our data, the SPSS version 20 was utilized. One-way analysis of variance (ANOVA) was used to compare the means with a post hoc test (LSD) demonstrating significance between groups. The Chi-square test was performed to show the significance between qualitative variables among groups. Pearson’s correlation test was performed to show the degree of association between quantitative variables.

## Results

In this study, we prepared a collagen II-induced arthritis model to evaluate the anti-inflammatory effects of pyrazole derivatives M1E and M1G and declare their mechanism of action.

### Effect of pyrazole derivatives M1E and M1G treatment on the severity of arthritis scores

**Fig. 1:**
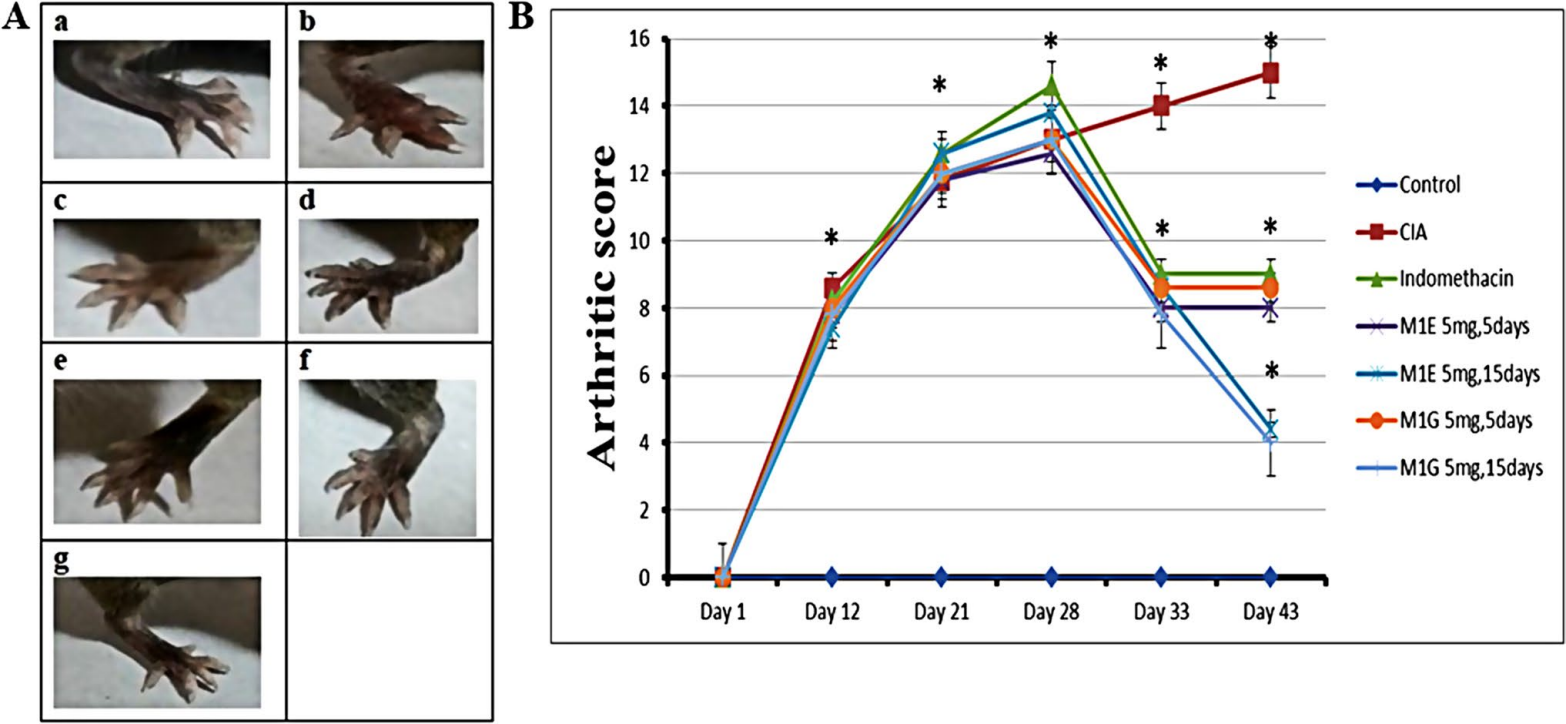
Ankle joints and hind paws of the studied animal groups. **A** Representative photographs of ankle joints and hind paws: (a) healthy control, (b) arthritis-induced model, (c) 1 mg/kg indomethacin-treated mice, (d) M1E for 5 days, (e) M1E for 15 days, (f) M1G for 5 days, and (g) M1G for 15 days of treatment. **B** Arthritis score outcomes of all groups. Results are given as mean ± SD (*n* = 5) and analyzed by one-way ANOVA followed by post hoc test (LSD). The mean difference between groups was considered statistically significant when *p* < 0.05

To assess the anti-inflammatory impact of pyrazole derivatives M1E and M1G on RA, we evaluated the arthritis score after treatment with various doses and time points of M1E and M1G compared to the indomethacin. Mice were handled in the manner that is outlined in the experimental section in an attempt to prepare collagen type II arthritis. Representative images of ankle joints and hind limb changes for each group are displayed in Fig. 1A. The control group showed no signs of swelling or redness throughout the study. In contrast, the arthritic mice showed joint swelling, redness, and lack of appetite, which worsened as the inflammation progressed up the limb. However, the pyrazole compounds M1E and M1G showed profound improvement in these symptoms. According to the evaluation criteria, the arthritis score in the CIA model group was significantly (*p* < 0.001) greater than that of the control group from day 12 onwards and continued to rise. Nearly all mice showed the typical symptoms by day 28, and their score peaked on day 43 (15 ± 0.71). On day 33, mice who received indomethacin 1 mg/kg for 5 days as a conventional medication reported a substantial reduction (9 ± 1.58) in the arthritic score (*p* < 0.001) relative to arthritic control mice, as shown in Fig. 1B. The arthritis score in M1E 5 mg/kg for 5 days (8 ± 1.58) and M1gG 5 mg/kg for 5 days (8.6 ± 1.14) groups was significantly (*p* < 0.001) less than that of the arthritic model group; however, this decrease in score in both groups was not significant when compared to the indomethacin group (*p* = 0.27 and 0.66, respectively). On day 43, the arthritis score in M1E (4.4 ± 0.89) and M1G (4 ± 1.73) groups treated for 15 days was significantly (*p* < 0.001) low when compared to the arthritic mice group and indomethacin group (*p* < 0.001). The decrease in the arthritic score was slightly high, with the M1G 5 mg/kg for the 15-day treatment group, as shown in Fig. 1B.

### Impact of M1E and M1G on mRNA expression of the studied inflammatory genes

To explore the effect of pyrazole-based compounds on genes of the inflammatory pathways, we examined the mRNA expression of pro-inflammatory markers p38MAPK, COX-2, IL1β, MMP3, and TNF-α genes in DBA/1J mice ankle joint homogenates using real-time PCR.

**Fig. 2:**
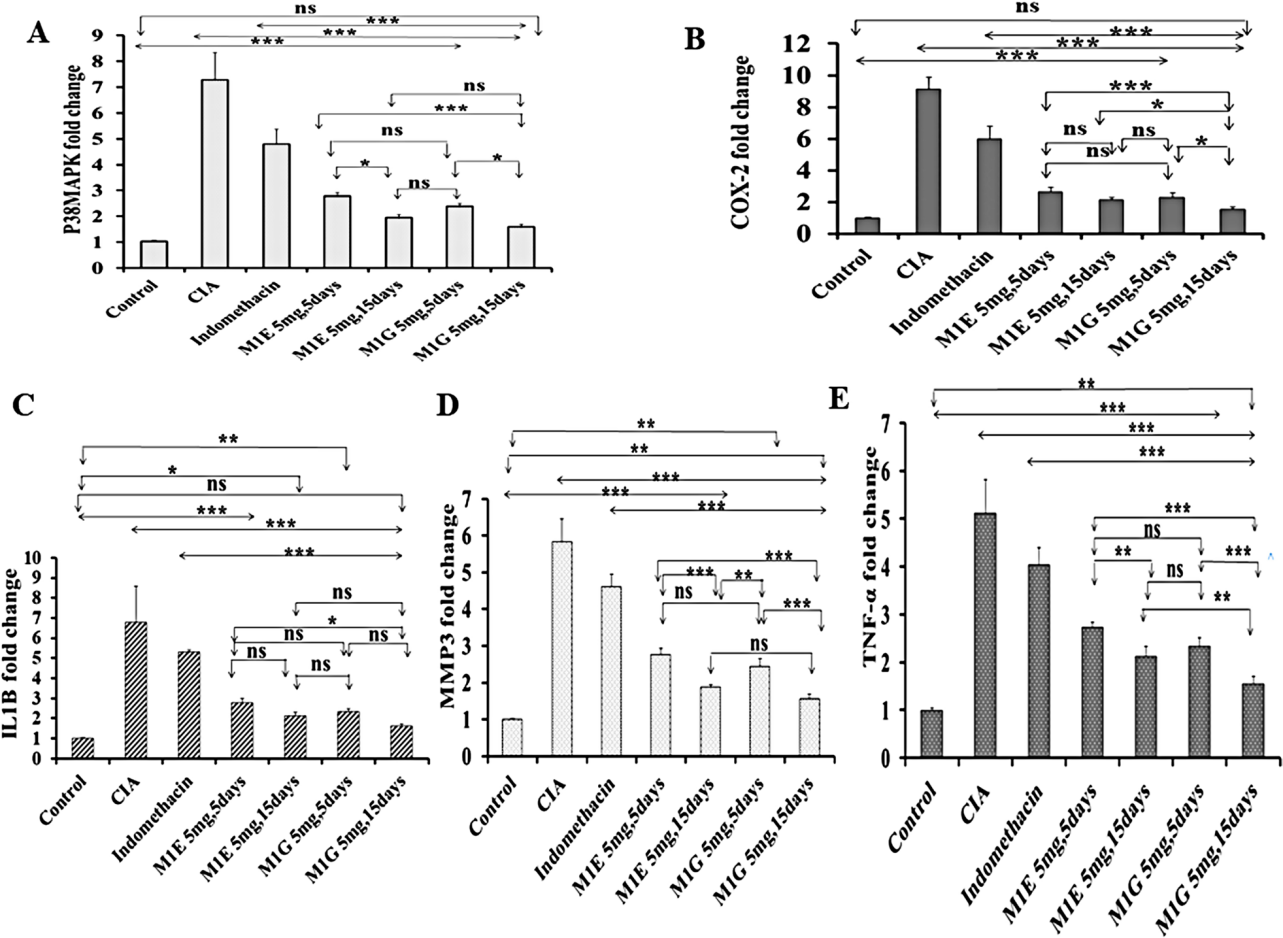
Effect of M1E and M1G on mRNA expression of the studied inflammatory genes. **A** RQ p38MAPK, **B** RQ COX-2, **C** RQ IL1β, **D** RQ MMP3, and **E** RQ TNF. Data is expressed as mean ± SD. Values were analyzed using one-way ANOVA followed by a post hoc test (LSD). The mean difference between groups was considered statistically significant when *p* < 0.05. High significance (***) when *p* < 0.001, moderate significance (**) when *p* < 0.01, and non-significant (ns) when *p* > 0.05

The gene expression of p38MAPK was significantly (*p* < 0.001) increased in the arthritis group (7.27 ± 1.06) when compared to healthy mice (1.00 ± 0.06). However, after 5 days of treatment with M1E and M1G, there was a significant (*p* < 0.001) downregulation of p38MAPK gene expression (2.75 ± 0.17 for M1E and 2.35 ± 0.15 for M1G) compared to indomethacin treatment (4.78 ± 0.59). More reduction in p38MAPK gene expression occurred after 15 days of treatment with M1E (1.92 ± 0.16) and M1G (1.56 ± 0.14) in comparison with 5 days (*p* < 0. 01 and 0.013, respectively). M1G demonstrated a more restoring effect on p38MAPK than M1E for an equal duration. Nonetheless, these changes were not statistically significant (*p* = 0.196 for M1E and M1G after 5 days and 0.24 after 15 days), as shown in Fig. 2A.

The arthritic group showed a significantly (*p* < 0.001) high mRNA expression of COX-2 (9.13 ± 0.73 fold) compared to the normal control group (1.00 ± 0.06). On the other hand, treatment with M1E (2.62 ± 0.30 fold) and M1G for 5 days (2.29 ± 0.31 fold) significantly (*p* < 0.001) reduced COX-2 gene expression in arthritic mice in comparison to indomethacin (5.99 ± 0.77 fold). The gene expression of COX-2 was significantly (*p* < 0.011) low after 15 days of treatment with M1G (1.53 ± 0.15) compared to 5 days of treatment, while it was non-significantly (*p* = 0.1) low after 15 days of treatment with M1E (2.15 ± 0.11 fold). M1G showed a higher ability to regenerate COX-2 gene expression than M1E throughout the same duration (*p* = 0.24 for 5 days and *p* = 0.034 for 15 days), as shown in Fig. 2B.

The arthritic control group had a significantly (*p* < 0.001) higher IL1β gene expression (6.78 ± 1.8 fold) compared to the healthy control (1.00 ± 0.03). After administering M1E (2.78 ± 0.22 fold) and M1G to arthritic mice for 5 days (2.32 ± 0.18 fold), the gene expression of IL1β was significantly reduced (*p* < 0.001) in comparison to indomethacin therapy (5.28 ± 0.13 fold). After 15 days, the mRNA expression of IL1β was insignificantly reduced with M1G (1.61 ± 0.12) and M1E (2.12 ± 0.17 fold) compared to 5 days of treatment (*p* = 0.123 and 0.143, respectively). M1G had a greater impact on restoring IL1β gene expression than M1E throughout the same duration. Still, the difference was not statistically significant (*p* = 0.302 for 5 days and 0.265 for 15 days, respectively), as presented in Fig. 2C.

A significant (*p* < 0.001) upsurge in MMP3 expression was observed in the arthritic group (5.83 ± 0.62), which was significantly (*p* < 0.001) reduced after 5 days of treatment with M1E (2.76 ± 0.18) or M1G (2.44 ± 0.21) in comparison with indomethacin therapy (4.60 ± 0.36). After 15 days of therapy, the expression of MMP3 mRNA decreased significantly (*p* < 0.001) compared to the 5 days of treatment with M1E and M1G (1.88 ± 0.066 and 1.56 ± 0.13, respectively). M1G was more effective than M1E in regenerating MMP3 expression in the arthritic mice over a similar period (*p* = 0.098 for 5 days and 0.104 for 15 days, respectively), as shown in Fig. 2D.

A remarkably elevated gene expression (*p* < 0.001) of TNF-α was evidenced in the arthritic group (5.12 ± 0.69 fold) that declined significantly (*p* < 0.001) in arthritic mice treated with M1E for 5 days (2.73 ± 0.10 fold) and M1G for 5 days (2.35 ± 0.17 fold) in comparison with indomethacin (4.05 ± 0.35 fold). The expression of TNF-α mRNA was significantly reduced after 15 days of M1E (2.12 ± 0.22 fold, *p* < 0.005) and M1G treatment (1.56 ± 0.14, *p* < 0.001) compared to a 5-day therapy. The TNF-α mRNA expression was more significantly (*p* < 0.009) downregulated in M1G than in M1E for 15 days but insignificantly for 5 days (*p* = 0.72) (Fig. 2E).

### Effect of M1E and M1G on protein expression of p38MAPK and COX-2

**Fig. 3:**
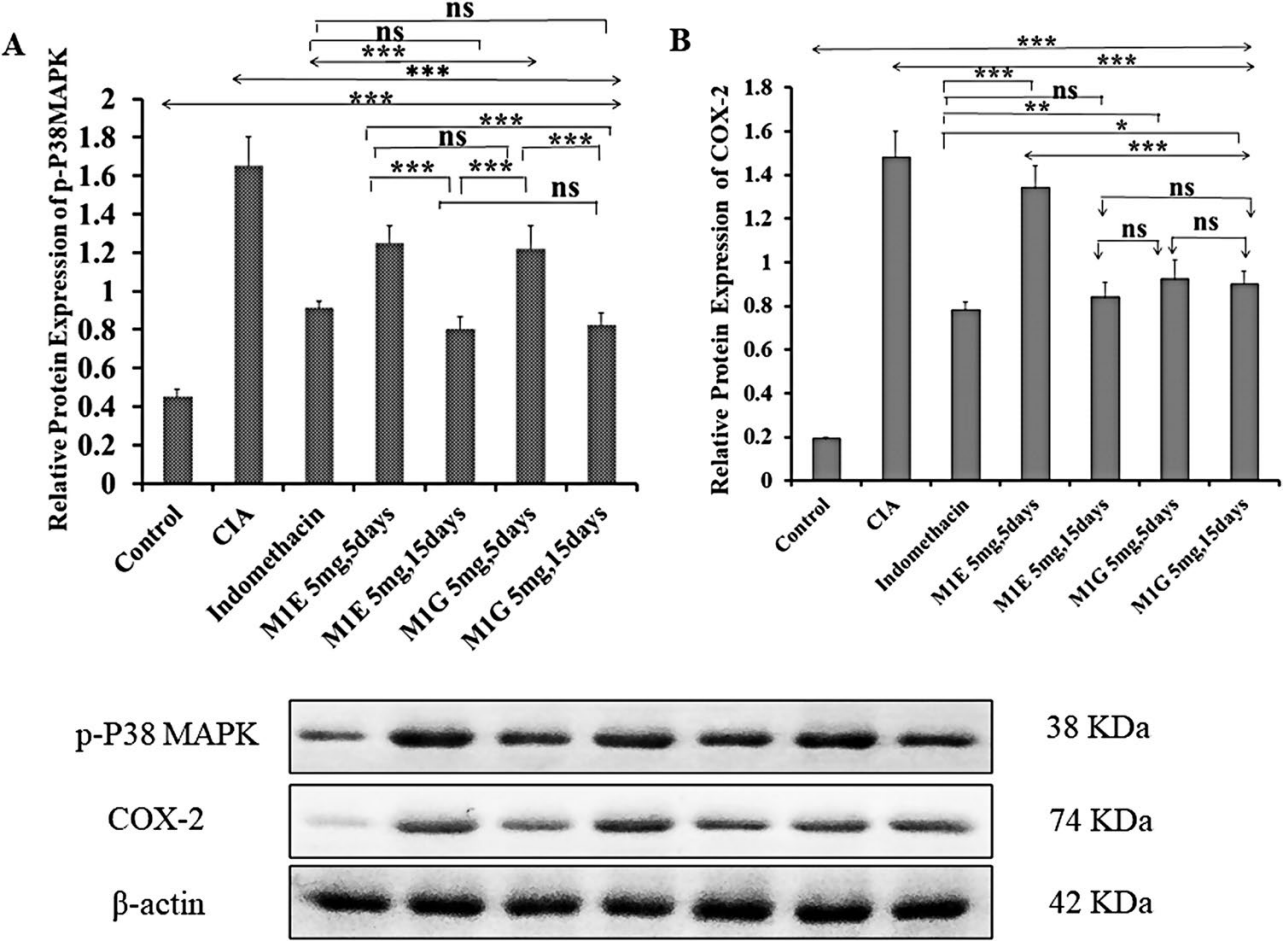
Effects of M1E and M1G on the protein expression of **A** p-p38 MAPK and **B** COX-2. Data is expressed as mean ± SD. Values were analyzed using one-way ANOVA followed by a post hoc test (LSD). The mean difference between groups was considered statistically significant when *p* < 0.05. High significance (***) when *p* < 0.001, moderate significance (**) when *p* < 0.01, and non-significant (ns) when *p* > 0.05

**Fig. 4:**
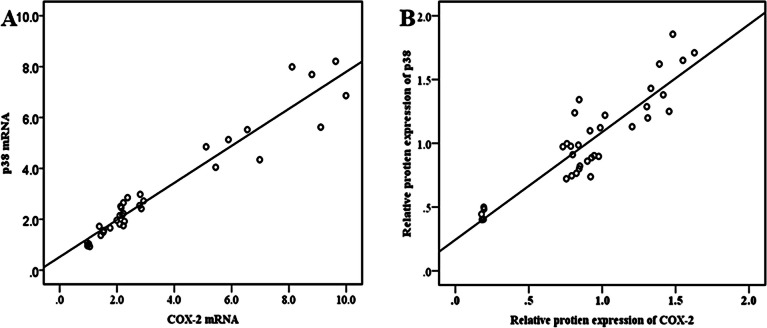
Correlation between p38 and COX-2 expression. **A** mRNA expression as measured by qRT-PCR, **B** protein expression as quantified from western blot

To confirm the involvement of p38 and COX-2, we evaluated their protein expression using western blotting. Our study focused on the phosphorylated p38 MAP kinase since the phosphorylation of p38 MAP kinase leads to transcription initiation by binding to regulatory sites on DNA, in turn, leads to the activation of pro-inflammatory cytokines such as TNF-α and IL-1, which play a central role in the pathogenesis of RA (Kim et al. 2010). The protein level of phosphorylated p38MAPK was significantly (*p* < 0.001) higher in the arthritic model group (1.65 ± 0.15) than in the healthy control group (0.45 ± 0.04). After treatment, the level of phosphorylated p38MAPK protein in the indomethacin group (0.91 ± 0.04), M1E for 5 days (1.25 ± 0.09), and M1G 5 mg/kg for 5 days (1.22 ± 0.12) groups were significantly (*p* < 0.001) reduced in comparison with arthritic mice. M1E (0.8 ± 0.07) and M1G (0.82 ± 0.07) were more significantly (*p* < 0.001) effective in suppressing p38MAPK protein expression for 15 days compared to 5 days as shown in Fig. 3A. Simultaneously, protein expression of COX-2 was significantly (*p* < 0.001) upregulated in the model group (1.48 ± 0.12) relative to the healthy control group (0.19 ± 0.007). Following treatment, the indomethacin group (0.78 ± 0.04), M1E for 5 days (1.34 ± 0.1), M1G 5 mg/kg for 5 days (0.92 ± 0.09) groups showed a significant (*p* < 0.001) reduction in COX-2 protein levels compared to the model group. The inhibitory effect of the pyrazole derivatives M1E and M1G on COX-2 protein expression was significantly (*p* < 0.001) greater after the 15 days of treatment compared to 5 days (0.84 ± 0.07 and 0.9 ± 0.06, respectively) as presented in Fig. 3B. Pearson’s correlation analysis showed a significant positive correlation between p38 MAPK and COX-2 mRNA gene expressions (*r* = 0.963, *p* < 0.001) and protein expressions (*r* = 0.9, *p* < 0.001), as shown in Fig. 4.

### Impact of M1E and M1G on oxidative stress markers

**Fig. 5:**
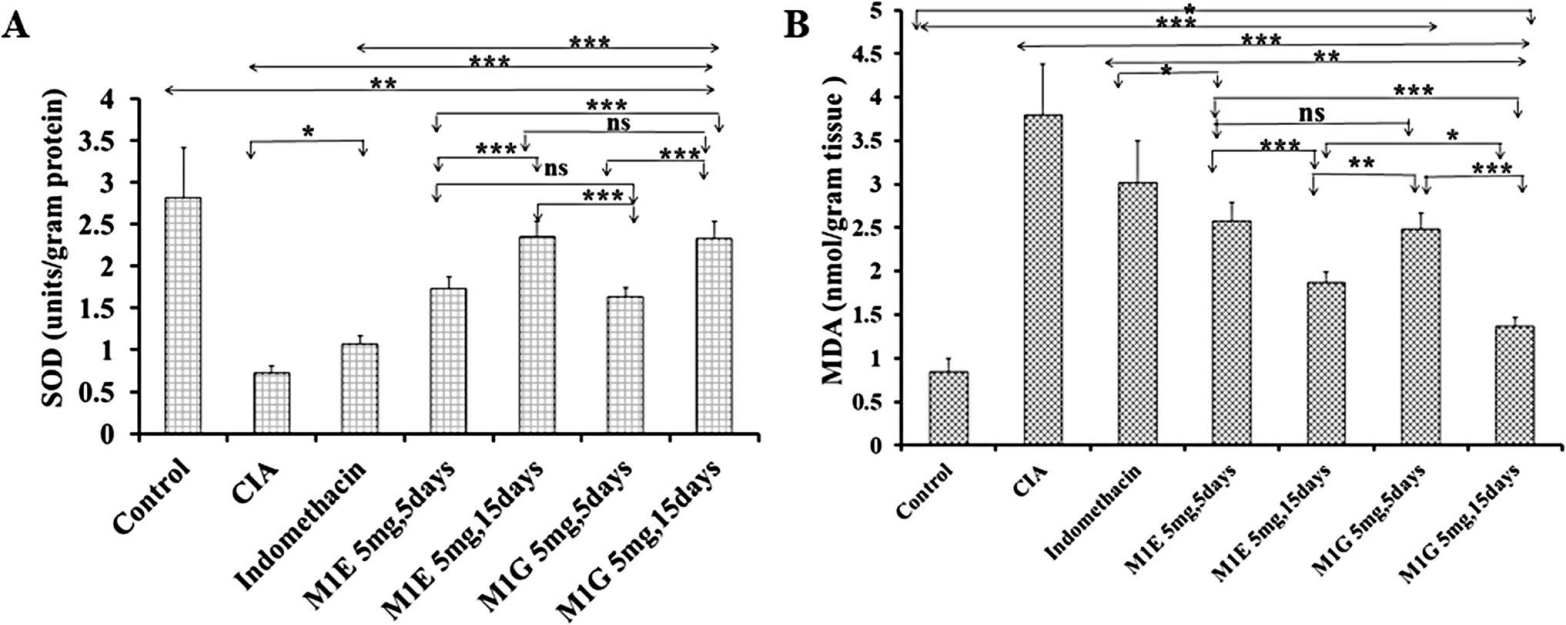
Effect of M1E and M1G on oxidative stress markers. **A** Activity of SOD, **B** concentration of MDA. Data are expressed as mean ± SD. Values were analyzed using one-way ANOVA followed by a post hoc test (LSD). The mean difference between groups was considered statistically significant when *p* < 0.05. High significance (***) when *p* < 0.001, moderate significance (**) when *p* < 0.01, and non-significant (ns) when *p* > 0.05

To evaluate the impact of M1E and M1G on the oxidative stress in arthritic mice, key biomarkers, including superoxide dismutase and malondialdehyde in paw tissue homogenates of DBA/1J mice, were measured in this study. MDA, which indicates lipid peroxidation, was measured in nmol/gram tissue, while SOD activity was measured in units/gram protein. The MDA levels significantly (*p* < 0.001) increased (3.8 ± 0.58), while SOD activity significantly (*p* < 0.001) reduced (0.73 ± 0.08) in arthritic mice in comparison to the normal control group (0.84 ± 0.16 and 2.82 ± 0.59, respectively). Both M1E and M1G treatments, which are given at 5 mg/kg for 5 days, significantly (*p* < 0.001) attenuated the concentration of MDA (2.58 ± 0.21 and 2.48 ± 0.19, respectively) as well as significantly (*p* < 0.001) increased SOD activities in arthritic mice (1.74 ± 0.13 and 1.64 ± 0.097, respectively) as shown in Fig. 5. This effect was comparable to that of indomethacin (3.02 ± 0.48 and 1.08 ± 0.09, respectively). Our study showed that longer duration of treatment with M1E and M1G resulted in considerably significant (*p* < 0.001) restoration of MDA (1.87 ± 0.12 and 1.37 ± 0.1, respectively) and SOD (2.36 ± 0.17 and 2.34 ± 0.19, respectively) levels in comparison with indomethacin.

### Pathological assessment

**Fig. 6:**
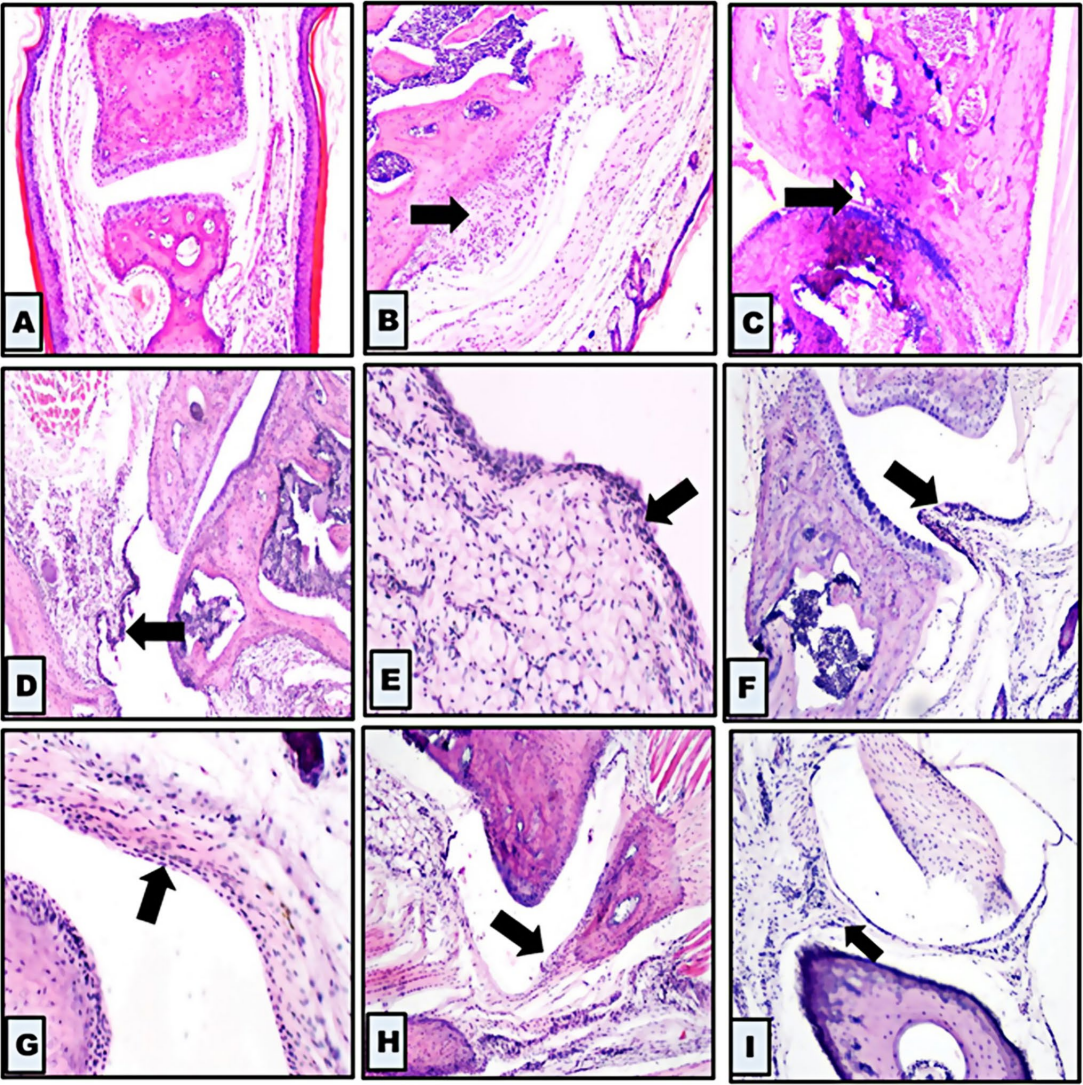
Histopathological changes in different groups. **A** A section from the control group showing joint within normal histological limits [no synovitis] (100 ×). **B**, **C**, **D** Images are sections from the collagen-induced arthritis group [high-grade synovitis] where **B** shows increased density of the resident cells (arrow), **C** shows fibrous ankylosis of joint cartilages (arrow), and **D** shows increased inflammatory infiltrate (arrow) (200 ×). **E** A section from the group treated with indomethacin for 5 days shows a mild enlargement of the synovial lining cell layer [low-grade synovitis] (100 ×). **F** Section from the group treated with E 5 mg for 5 days showing mild enlargement of the synovial lining cell layer [low-grade synovitis] (100 ×). **G** Section form group treated with E 5 mg for 15 days showing mild enlargement of the synovial lining cell layer [low-grade synovitis] (100 ×). Sections from the group treated with G 5 mg for 5 days (H) and from the group treated with G 5 mg for 15 days (I) showed histological features comparable to normal [no synovitis] (100 ×)

**Fig. 7:**
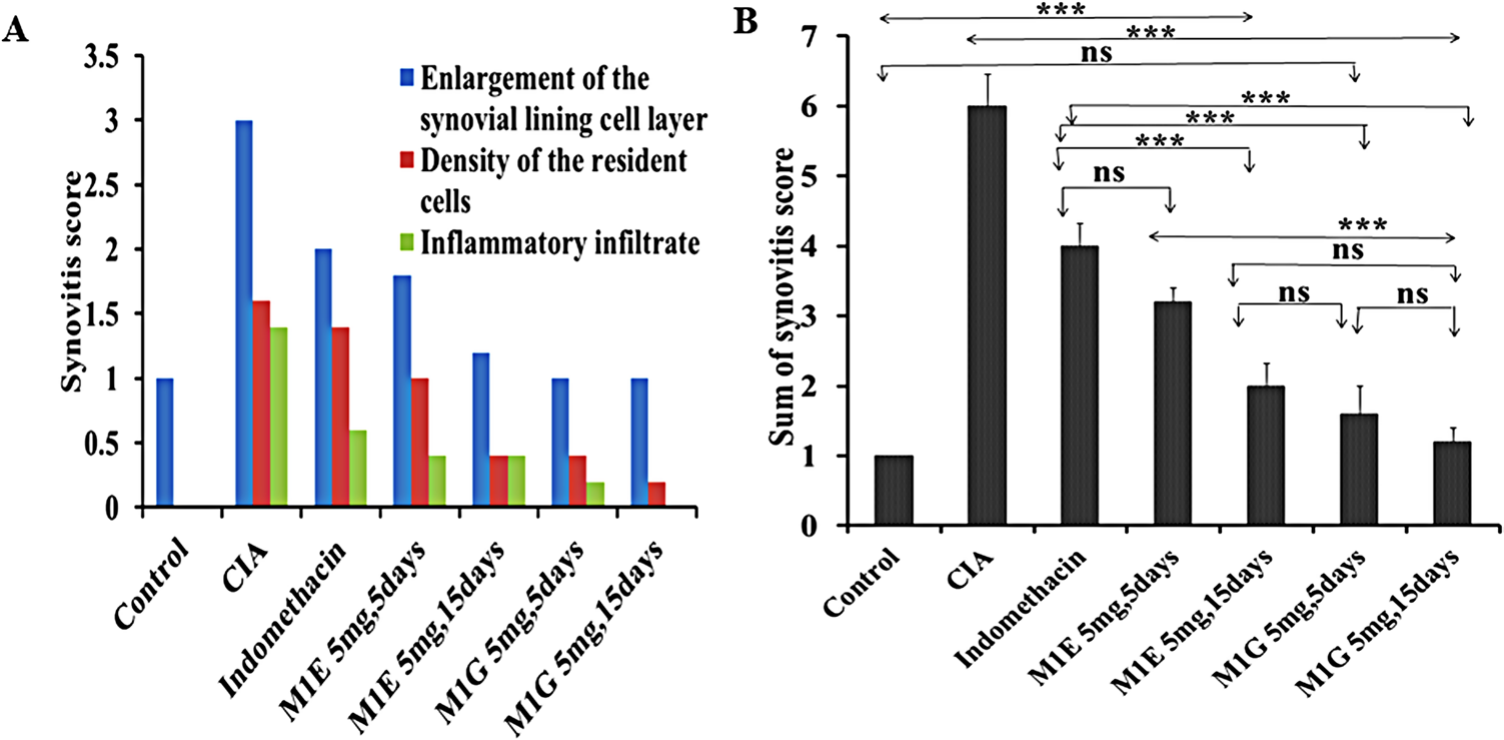
Effect of M1E and M1G on the pathological changes of the hind paw and knee joints. **A** Histopathological parameter scores of synovitis (synovial lining cell layer, density of the resident cells, and inflammatory infiltrate). **B** The sum of synovitis scores in the studied groups. Data is expressed as mean ± SD. Values were analyzed using one-way ANOVA followed by a post hoc test (LSD)

Additional proof to support the inhibitory impact of pyrazole derivatives M1E and M1G on collagen II arthritis was acquired through pathological assessment of joints. At the end of the experiment, the knee and ankle joints of the experimental mice were resected, fixed, and decalcified. The results of joint histopathology at the end of 43 days of the study revealed that all control group mice (100%) had no synovitis and all joints had normal histological features, ordinary cartilage covering appearance, and joint space. In the RA group, RA was observed to be well-developed, as shown in Fig. 6, showing fibrous ankylosis of joint cartilages (3 ± 0.00), increased density of the resident cells (1.6 ± 0.24), and increased inflammatory cell infiltration (1.4 ± 0.24) as shown in Fig. 7A, and the three parameters were significantly worse in the collagen-induced arthritis group (RA vs control, *p* < 0.001). All mice in the collagen-induced arthritis group (100%) had high-grade synovitis (synovitis score ≥ 5 points). In the indomethacin group, 20% of animals had high-grade synovitis, and 80% exhibited a therapeutic effect with low-grade synovitis (*p* < 0.001 vs collagen-induced arthritis group). However, in the 5-day treatment group with M1E 5 mg/kg, all mice (100%) exhibited low-grade synovitis with only mild synovial lining cell layer enlargement. Besides, there was no significant difference compared to the indomethacin group (*p* = 0.072), as shown in Fig. 7B. On the other hand, when treated with M1E (5 mg/kg) for a longer duration of 15 days, 80% of the mice in this group had low-grade synovitis with mild enlargement of the synovial lining cell layer, and 20% showed no synovitis at all (*p* < 0.001 when compared to indomethacin group). M1G 5 mg/kg after 5- and 15-day treatment periods effectively improved RA pathologic features with a greater inhibitory effect than the indomethacin group (*p* < 0.001 for both groups compared to the indomethacin group). In the case of 5 mg/kg M1G, 40% had low-grade synovitis, and 60% had no synovitis after 5 days, while 20% had low-grade synovitis and 80% had no synovitis after 15 days (Table 1).

**Table 1:** Degree of synovitis among different study groups using Chi-square test

Groups	Score interpretation			Total
	No synovitis	Low-grade synovitis	High-grade synovitis	
Control	5 (100%)	0 (0%)	0 (0%)	5 (100%)
CIA	0 (0%)	0 (0%)	5 (100%)	5 (100%)
Indomethacin	0 (0%)	4 (80%)	1 (20%)	5 (100%)
M1E 5 mg/kg, 5 days	0 (0%)	5 (100%)	0 (0%)	5 (100%)
M1E 5 mg/kg, 15 days	1 (20%)	4 (80%)	0 (0%)	5 (100%)
M1G 5 mg/kg, 5 days	3 (60%)	2 (40%)	0 (0%)	5 (100%)
M1G 5 mg/kg, 15 days	4 (80%)	1 (20%)	0(0%)	5 (100%)

## Discussion

Pyrazole compounds have been synthesized with various pharmacological activities, including antibacterial, anti-tuberculosis, anticancer, antioxidant, antifungal, and antiviral agents (Ansari et al. 2017). Furthermore, their medical potential for managing inflammation led to the creation of safer anti-inflammatory drugs like celecoxib, which have been introduced to the clinic (Marzouk et al. 2021).

It is a well-accepted fact that animal models have limitations in reflecting the complexity of human diseases. For instance, the CIA in mice is one of the most commonly used models to mimic human RA. It is often used for preclinical testing in rodents because it shares similarities with RA in humans in pathology (Saleem et al. 2020). Collagen II (CII) is considered to be the major protein in joint cartilage. Previous studies indicate that the immune system will be triggered when the CII of different species is present in the joint cartilage, leading to the production of anti-CII antibodies by B cells and the development of the CIA (Li et al. 2014). The objective of the current research was to investigate the potential of two pyrazole compounds, M1E and M1G, in reducing polyarthritis in DBA/1J mice and to determine the mechanism of action involved.

In this study, we noticed that the inflammation started on day 12 and progressively worsened by day 28, continuing to day 43, owing to the ongoing swelling and redness. The pyrazole compounds, M1E and M1G, significantly (*p* < 0.001) reduced the arthritis score index after 5 days of treatment compared to the arthritic and indomethacin groups. The M1E and M1G groups showed further significant (*p* < 0.001) reduction in arthritis score after 15 days of treatment compared to that of 5 days of treatment and to the indomethacin group as evidenced by the decrease in paw swelling and redness, suggesting a reduction in disease progression. Additionally, the M1G group that received a 5 mg/kg dosage for 15 days showed the greatest reduction in score. Our findings were parallel with a previous study by Bekhit et al. (2022), which concluded that rats treated with AD732, another pyrazole derivative, experienced significant suppression in carrageenan-induced rat paw edema formation compared to indomethacin.

Induction of arthritis in animals results in increased levels of pro-inflammatory mediators contributing to the progression of arthritis (Kim and Moudgil 2017). Additionally, the deformation of bone and cartilage along with severe inflammatory conditions, and oxidative injuries, exacerbates the severity of the disease and leads to stimulation of various pro-inflammatory mediators such as TNF-α, COX-2, and IL1β both in joint tissues and serum. TNF-α causes the production and maturation of B and T lymphocytes (Geng et al. 2018). The IL1β activates osteoclasts, which contribute to joint and bone damage. The increased levels of IL1β and TNF-α stimulate synovial fibroblasts, chondrocytes, and osteoclasts to accumulate tissue-destructing MMPs. Also, the increased generation of pro-inflammatory mediators further triggers the expression of oxidative stress markers, leading to enhanced inflammation and oxidative injuries (Zhang et al. 2020).

The IL1β is accountable for joint damage and synovitis by releasing ROS and proteolytic enzymes (Saleem et al. 2020). Moreover, these pro-inflammatory cytokines stimulate chemokines, which attract neutrophils and monocytes towards inflamed joints (Voon et al. 2017). To prevent bone and cartilage destruction, it is important to block pro-inflammatory cytokines like TNF-α, which are involved in gene expression of matrix metalloproteinase (MMPs) (Srirangan and Choy 2010). In this study, we found a significant (*p* < 0.001) increase in TNF-α and IL1β gene expression in the arthritic group in comparison with normal controls, and this is in agreement with Zhang et al. (2020); interestingly, treatment with M1E and M1G led to a significant (*p* < 0.001) reduction in their expression in the polyarthritis’ mice in comparison to the indomethacin. These results aligned with the findings of Ragab et al. (2023), whose study showed that serum levels of IL1β and TNF-α measured by ELISA were significantly reduced after treatment of arthritic mice with pyrazole-based derivatives 7a and 7b, confirming the anti-inflammatory activities of these compounds.

MMP3 is a proteolytic enzyme that contributes to the destruction of bone and the degradation of cartilage components. We found that arthritic mice had significantly (*p* < 0.001) higher levels of MMP3 mRNA expression compared to healthy mice, and this agrees with the findings of Li et al. (2014). Galil et al. (2016) reported that high levels of MMP3 are strongly associated with disease progression and can act as an early warning sign of joint damage, suggesting that MMP3 could be a valuable prognostic marker for monitoring RA activity. Our results demonstrate that pyrazole M1E and M1G therapy significantly (*p* < 0.001) reduced the MMP3 mRNA expression in arthritic mice which may help stop cartilage degradation.

Mitogen-activated protein kinases (MAPKs) are crucial intracellular signaling molecules that regulate the inflammatory response. Recent studies indicate that MAPKs in macrophages are highly activated and involved in RA pathogenesis (Jiang et al. 2019). The MAPK family comprises three subfamilies: ERK, p38, and JNK. P38 MAPK is an important signaling molecule of inflammation responsible for synovial inflammation, promoting the secretion of inflammatory cytokines in activated macrophages and the synthesis of RA collagenase. When p38 MAPK is phosphorylated, it activates TNF-α and IL-1 at the transcriptional level (Genovese et al. 2011). As a result, p38 MAP kinase is involved in synovial inflammation, cartilage degradation, inflammatory bone loss, and angiogenesis (Patel and Pundarikakshudu 2016). In this study, we observed a significant (*p* < 0.001) increase in the expression of p38MAPK mRNA and phosphorylated p38MAPK protein levels in ankle tissue homogenates of arthritic mice compared to healthy mice. However, treatment with M1E and M1G significantly decreased (*p* < 0.001) in both the p38MAPK mRNA and the phosphorylated p38MAPK protein expression levels in the arthritic mice. Somakala et al. (2016) found that compound 4a, based on pyrazole, had a favorable orientation within the active binding site of p38 MAPK, resulting in a relatively higher docking score. Studies have demonstrated that inhibiting p38 MAPK can also suppress the production of TNF-α and IL-1. Additionally, reducing p38 MAPK activity has been shown to decrease paw swelling and joint damage in rat models of RA (Genovese et al. 2011). These findings suggest that pyrazole compounds may impact the production of inflammatory mediators and cytokines by stopping the signaling pathway of p38.

COX-2 is a major inflammatory enzyme that generates prostaglandins like PGE2, which elicit pain and inflammation in RA. The liberation of PGE2 from chondrocytes and synovial fibroblasts was instigated by pro-inflammatory cytokines like TNF-α and IL1β (Lee et al. 2019). Yang et al. (2017) found that COX-2 is minimally expressed in normal cells and tissues and is only produced when induced. Elevated COX-2 expression is linked to synovial inflammation in arthritis. According to our study, the arthritic mice had a significantly (*p* < 0.001) higher mRNA and protein expression of COX-2 compared to the normal healthy group. This finding is parallel with a previous study by Saleem et al. (2020) who observed a similar increase in COX-2 mRNA expression in the CFA-stimulated arthritic rats. In contrast, treatment of arthritic mice with M1E and M1G significantly (*p* < 0.001) restored the increased mRNA and protein expression levels of COX-2. This finding is supported by Bekhit et al. (2022), who found that another pyrazole-based compound “AD732” strongly inhibits COX-2 enzymatic activity indicating that pyrazoles have the potential to inhibit the inflammatory responses in the body. The p38 MAPK was found to be involved in the regulation of COX-2 gene expression (Endale et al. 2013). In the current study, we found a strong association between the gene expressions of p38 MAPK and COX-2 mRNA, with a significant (*p* < 0.001) strong positive correlation (*r* = 0.963). The p38 MAPK and COX-2 protein expression also showed a significant (*p* < 0.001) strong positive correlation (*r* = 0.9).

The pathologic process of arthritis is greatly influenced by oxidative stress. Neutrophils and macrophages produce excessive amounts of reactive oxygen species (ROS) that contribute to joint damage via upregulating matrix metalloproteinases and activating osteoclasts (Mateen et al. 2016). The increased levels of ROS, COX-2, and lipid peroxidation cause an imbalance of antioxidant mechanisms leading to the loss of the antioxidant protection systems and endangering the intracellular antioxidant enzymes, particularly SOD, which normally detoxify the superoxides and peroxides (Quiñonez-Flores et al. 2016). We found that arthritic mice had significantly (*p* < 0.001) decreased SOD levels, and this is consistent with Saleem et al. (2020). MDA is used as an indicator of oxidative stress damage (Hosseini et al. 2018). We found an elevated MDA level in collagen-induced oxidative stress mice due to lipid peroxidation of membranes, and this finding was similarly reported by Aloke et al. (2022). In contrast, treatment of arthritic mice by M1E and M1G significantly decreased the levels of MDA and restored the morphology of the cell membrane, which may also be due to their ability to increase the levels of SOD, indicating the anti-oxidative effect of pyrazole compounds. In fact, after 15 days of treatment, M1E and M1G were more effective in reducing oxidative stress in polyarthritic mice compared to 5 days of treatment and indomethacin. These findings are consistent with a study by Somakala et al. (2016), which found that pyrazole compounds 4a–4c, 4e, and 4g significantly reduced MDA levels measured in rats with carrageenan-induced hind paw edema. Overall, pyrazole derivatives are expected to reduce oxidative stress, a key mechanism for anti-arthritic effects, by regulating inflammatory cytokines and COX-2 in arthritis.

CIA is well characterized by synovial hyperplasia, synovitis, pannus formation, deterioration of cartilage, and bone erosion in the joint (Endale et al. 2013). In the present study, the pathological assessment of joints showed that arthritic mice depicted fibrous ankyloses of joint cartilages, increased density of resident cells, and raised inflammatory cell infiltration with a high cumulative synovitis score ranging from 5 to 9 points. All mice in the arthritic group had high-grade synovitis (100%) when compared to the control group (*p* < 0.001). On the contrary, the M1E and M1G treatment reduced synovial hyperplasia and density of resident cells in mice with CIA. After 5 days of treatment with M1E 5 mg/kg, 100% of mice had low-grade synovitis. After 5 days of treatment with M1G 5 mg/kg, 40% of animals had low-grade synovitis, and 60% had no synovitis. Longer treatment durations with M1E and M1G (5 mg/kg for 15 days) significantly reduced the severity of these pathological changes; as after 15 days of treatment with M1E, 80% of animals had low-grade synovitis, and 20% had no synovitis. After 15 days of treatment with M1G, 80% of mice had no synovitis, and 20% had low-grade synovitis. This study is the first to demonstrate that compounds M1E and M1G, which belong to the pyrazole family, may be able to decrease disease progression as well as induce remission by reducing chronic inflammation, controlling synovitis, and preventing joint destruction in comparison to indomethacin, a typical NSAID, particularly with prolonged treatment.

It is important to point out that pyrazole compound M1G is a little more potent than M1E in regulating inflammatory mediators in CIA mice and was able to fully reverse the arthritic changes in CIA mice, and administering either M1E or M1G once daily for a longer duration is more effective. Our study shows that M1E and M1G have significant anti-arthritic activity in the CIA mice model. They significantly reduced the arthritic score and the gene expression of pro-inflammatory cytokines such as p38 MAPK, COX-2, IL1β, MMP3, and TNF-α. Additionally, both derivatives significantly reduced the protein expression of phosphorylated p38MAPK and COX-2, ameliorated oxidative stress by reducing MDA and restoring enzymatic antioxidants, SOD, and improved the histopathological features of synovitis compared to indomethacin as a standard drug. The findings suggest that both M1E and M1G could be valuable anti-inflammatory and anti-oxidative agents for managing RA. However, the potential toxic effects of long-term use should be evaluated before clinical trials in comparison to other available drugs.

## Conclusion

The pyrazole derivatives, M1E and M1G, significantly reduced the arthritic score and the inflammatory cytokine expression, improved synovitis histopathology, and ameliorated oxidative stress in the CIA mice model via targeting p38MAPK and COX-2. However, further studies are needed to conduct trials on human subjects and determine their effects on other inflammatory signaling pathways involved in RA.

## Data Availability

The current study’s data is available from the corresponding author on reasonable request.
